# Plasma concentrations of soluble Fas receptors (Fas) and Fas ligands (FasL) in relation to CD4+ cell counts in HIV-1 positive and negative patients in Yaounde, Cameroon

**DOI:** 10.1186/1756-0500-5-322

**Published:** 2012-06-22

**Authors:** George M Ikomey, Marie-Claire Okomo-Assoumou, Julius Atashili, Martha T Mesembe, Bertha Mukwele, Emilia Lyonga, Agnes Eyoh, Peter M Ndumbe

**Affiliations:** 1 Center for the Study and Control of Communicable Diseases (CSCCD), Faculty of Medicine and Biomedical Sciences, University of Yaoundé 1, Yaoundé, Cameroon; 2 Faculty of Health Sciences, University of Buea, Buea, Cameroon

**Keywords:** Apoptosis, Fas, FasL, Immune activation, HIV, Cameroon

## Abstract

**Background:**

Though documented that HIV infection progresses with the depletion of CD4+ cells, the exact mechanisms by which these cell depletions occur are not clearly understood. This study aimed at evaluating the plasma levels of soluble Fas receptors and ligands in HIV-infected and uninfected patients in Yaounde, Cameroon, a population with a known diversity of HIV in whom this has not been previously assessed.

**Findings:**

In a cross-sectional study, 39 antiretroviral naïve HIV-1 positive and negative participants were recruited in Yaounde, Cameroon. CD4+ lymphocyte cell counts were quantified from whole blood using an automated FACScount machine (Becton-Dickinson, Belgium). Plasma samples obtained were analyzed for soluble Fas receptors and Fas ligands in both HIV-1 positive and negative samples using two different quantitative sandwich ELISA kits (Quantikine®, R&D Systems , UK).

Plasma levels of Fas receptors were higher in HIV-1 positive patients (median = 1486pg/ml IQR = 1193, 1830pg/ml) compared to HIV-negative controls (median = 1244pg/ml, IQR = 1109, 1325pg/ml), p-value <0.001. Plasma levels of Fas ligands were also higher in HIV-1 positive patients (median = 154pg/ml, IQR = 111, 203pg/ml) compared to HIV-negative controls (median = 51pg/ml, IQR = 32, 88pg/ml), p-value = 0.005. Plasma concentrations of soluble fas receptors and ligands tended to be negatively correlated with the CD4+ cell counts of HIV-positive patients; the correlation coefficients were -0.34 (value = 0.78) and-0.3 (p-value = 0.51) respectively.

**Conclusions:**

In this population of patients in Cameroon, plasma concentrations of Fas receptors and Fas ligands tend to be higher in HIV-positive patients. The Fas pathway of apoptosis may have a role in the depletion of CD4+ cell counts

## Findings

### Background

Infection with human immunodeficiency virus type 1 (HIV-1) is characterised by a gradual and progressive CD4+ T-helper cell depletion [1]. The Fas mediated mechanism of apoptosis during activation induced cell death (AICD) system is a key regulator of apoptosis in normal and malignant T cells [2,3]. Many studies have shown that the mechanism of T-cell receptor(TCR)-triggered activated induce cell death (AICD) in peripheral T-cell mediated via CD95 receptor/ligand interaction are up regulated and involved in this loss of CD4+ cells in HIV-1 infected patients[2,4]. All T-lymphocytes cells express the CD95 cell-surface receptor, a member of the TNF-R/NGT-R super family, which can induce apoptosis upon oligomerization with its ligand, resulting to CD4+ T-cell death [5].

In spite of these, the exact mechanisms by which these cell depletions occur are not clearly understood. Secondly it is not known if these mechanisms are universally similar irrespective of study population or viral type.

In this study we evaluated the plasma concentration of soluble Fas receptors and ligands in HIV-positive patients and compared them to HIV-negative controls. We also assessed the relationship between plasma concentrations of each of Fas receptors and ligands with CD4+ lymphocyte cell counts in HIV-1 positive patients in Cameroon, a population with a known diversity of HIV-1 in which these pathways have not been previously assessed.

## Methods

This study was reviewed and approved by Faculty of Medicine and Biomedical Sciences in Yaoundé, Cameroon.

Thirty-nine antiretroviral naïve HIV-1 positive and negative participants were recruited from the Centre for the Study and Control of Communicable Diseases (CSCCD) of the Faculty of Medicine and Biomedical Sciences in Yaoundé, Cameroon. After obtaining written consent, demographic data were recorded from each participant using a standardized questionnaire. Ten ml of blood was collected in EDTA anticoagulant tubes from each participant. The blood was then centrifuged at 12000rpm for five minutes and plasma separated and stored in two vials of 1.5ml each. The plasma was then analyzed for Fas receptor (CD95) and Fas ligands (CD95L) in both HIV-1 positive and negative samples using two different quantitative sandwich ELISA kits (Quantikine®, R&D Systems , UK). All samples were analysed based on the manufacturers’ guidelines. It is worth noting that this assays will detect any Fas ligands (or receptors) present in plasma and thus the sensitivity is not expected to be reduced because of the presence of receptor-ligand complexes.

Data were entered into a Microsoft Excel spreadsheet and analysed using STATA version 8 (STATA Corps Texas USA). Plasma levels of Fas receptor and Fas ligands levels in HIV positive and negative participants were compared using the Mann-Whitney U-Test. A spearman rank correlation coefficient was used to determine the correlation between each of Fas receptors and Fas ligands with CD4+ cell counts. These data are available, upon request, from the corresponding author.

## Results

**Figure 1:**
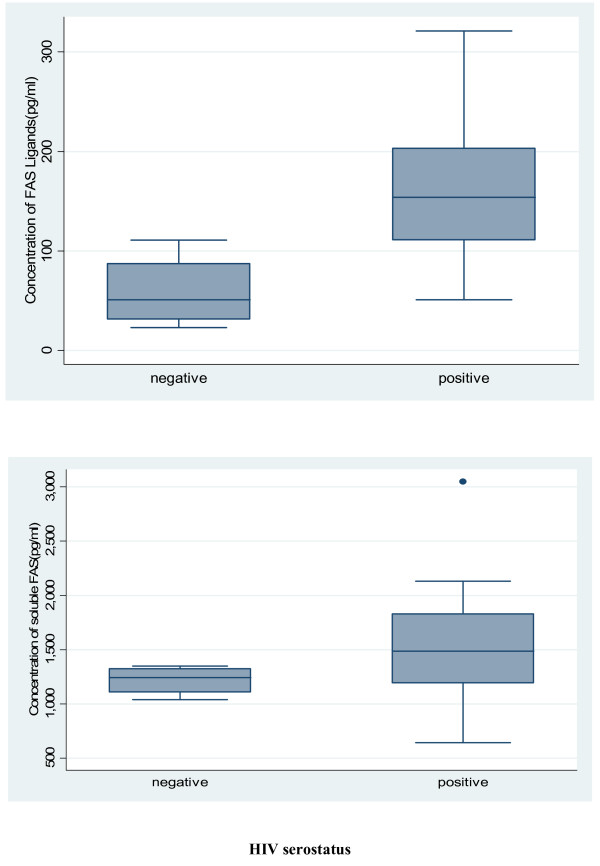
**Plasma concentration of Fas receptors (top panel) and Fas ligands (bottom panel) by HIV-1 status in 39 participants in Yaounde, Cameroon.** The middle horizontal lines represent the median concentrations of Fas ligands or Fas receptors.

Of the 39 participants , 19 (49%) were females and 27 (69%) were HIV-1 seropositive. Plasma levels of Fas receptors were higher in HIV-1 positive subjects with (median = 1486pg/ml, IQR = 1193, 1830pg/ml) compared to HIV-1 negative subjects (median = 1244pg/ml, IQR = 1109, 1325pg/ml) with a p-value of 0.005 (Figure 1). The levels of ligands in HIV-positive subjects (median = 154pg/ml, IQR = 111, 203pg/ml) were also higher compared to that in HIV-negative subjects (median = 51pg/ml, IQR = 32, 88pg/ml) with a p-value = 0.001(Figure 1).

**Figure 2:**
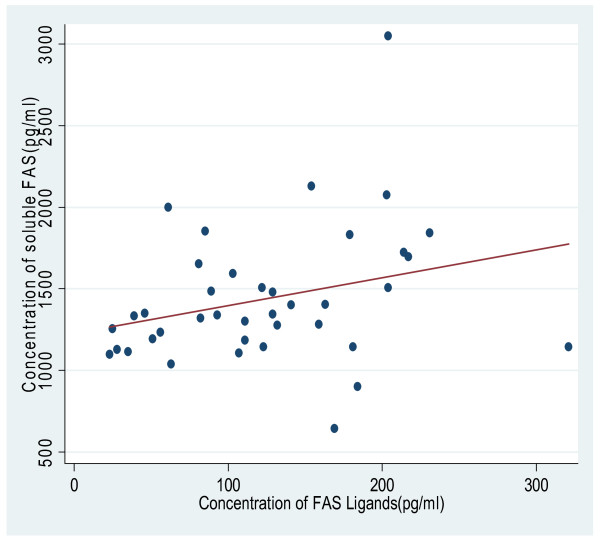
**Correlation between plasma levels of Fas receptors and Fas ligands in 39 participants in Yaounde, Cameroon.** The dots are the individual observations and the straight line is the line of best fit.

There was a weak correlation between plasma concentrations of Fas receptors and Fas ligands with a Spearman correlation coefficient of 0.32.(Figure 2).

**Figure 3:**
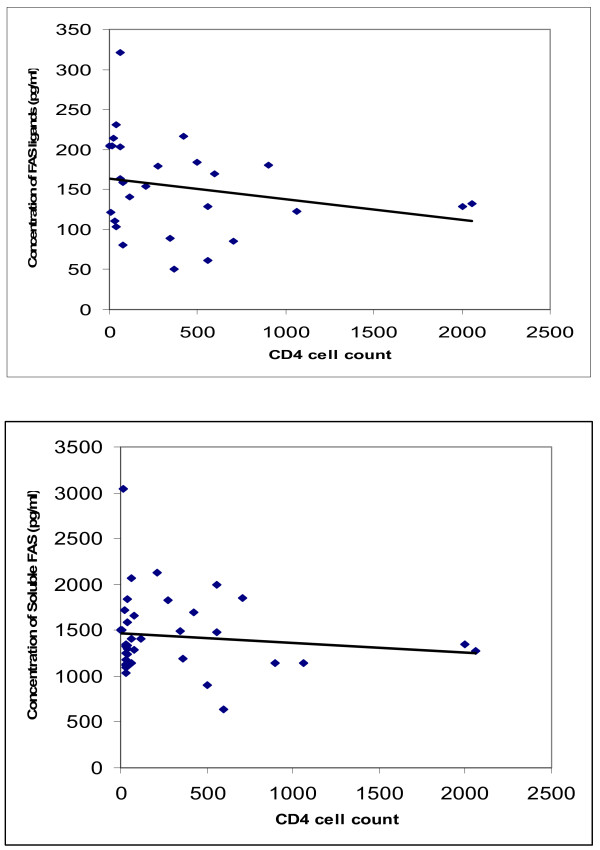
**Correlation between CD4+ cell counts and plasma levels of each of Fas receptors (upper panel) and Fas ligands (lower panel) in 27 HIV-positive participants in Yaounde, Cameroon.** For each panel, the dots are the individual observations and the straight line is the line of best fit.

Plasma concentrations of soluble Fas receptors and ligands were negatively (though not statistically significantly) correlated with the CD4+ cell counts of HIV-positive patients: the correlation coefficients were -0.34 (p-value = 0.78) and -0.3 (p-value = 0.51) respectively (Figure 3).

## Discussion

This study aimed at evaluating the plasma concentrations of soluble Fas receptors and ligands in relation to CD4+ cell depletion in a population with high HIV-1 genetic diversity. Our results show an increase in the levels of Fas receptors and ligands in HIV-1 positive patients compared to HIV-negative subjects. Some previous studies described an increase in Fas receptor expression in T-cells from HIV-1 infected children and adults [3-7]. In a similar study it was also shown that the membrane-bound Fas levels were higher in lymphocytes of HIV-1 positive compared to HIV-negative patients; an increase explained as resulting from a reduction in the pressure mounted on the normal immune system of HIV-uninfected cells when compared to infected cells [8-11]. The increase in Fas receptors and ligands in HIV-1 infected patients could be in concordance with Fas ligand shedding during T-cell activations, as most T-cells are under pressure from the virus [4].

We also found a depletion of the levels of CD4+ with increased Fas ligands and Fas receptors though this was not statistically significant.

While this study is strengthened by the inclusion of samples from Cameroon, with high HIV diversity, some limitations need to be considered. The relatively small sample size may have lowered the power to identify significant correlations between the Fas Ligands and CD4 counts. We did not analyze the correlation with viral load and HIV subtype. The cross-sectional nature of the study did not allow for establishing whether the time relationship between Fas ligands, the receptors and CD4+ lymphocyte accounts.

## Conclusions

Plasma concentration of Fas receptors and Fas ligand, could be involved in the decrease in CD4+ cells in HIV-1 patients. Further studies on the role of the Fas complex in HIV pathogenesis in this population with significant viral diversity are needed. These studies would assess the potential role of FAS ligands and receptors as indicators of disease progression and or response to antiretroviral therapy.

## Availability of supporting data

Data are available with corresponding author and can be made available upon request.

## Abbreviations

AICD, Activated induced cell death; CD, Cluster of differentiation; EDTA, Ethylene diamine tetra-acetic acid; ELISA, Enzyme linked immuno sorbent assays; IQR, Interquartile range; rpm, Revolution per Minute; TCR, T-cell receptors; TNF-R, Tumor necrotic Factor-R; UK, United Kingdom; USA, United State of America.

## Competing interests

The authors declare that they have no competing interests.

## Authors’ contributions

GMI conceived and designed the study, implemented sample collection and laboratory analysis, and wrote the first draft of manuscript. MCOA contributed in design and supervised laboratory implementation. JA participated in the design of the study, performed the statistical analysis and substantially revised first draft of manuscript. MM contributed in design and participated in laboratory implementation. BM contributed in design and participated in laboratory implementation. EL participated in laboratory implementation. AE participated in laboratory implementation. PMN contributed in design and supervised laboratory implementation. All authors read and approved the final manuscript.
